# Association Between *MTHFR* Polymorphisms and Congenital Heart Disease: A Meta-analysis based on 9,329 cases and 15,076 controls

**DOI:** 10.1038/srep07311

**Published:** 2014-12-04

**Authors:** Chao Xuan, Hui Li, Jin-Xia Zhao, Hong-Wei Wang, Yi Wang, Chun-Ping Ning, Zhen Liu, Bei-Bei Zhang, Guo-Wei He, Li-Min Lun

**Affiliations:** 1 Department of Clinical Laboratory, The Affiliated Hospital of Qingdao University, Qingdao, P.R China; 2 Department of Medical Ultrasonics, The Affiliated Hospital of Qingdao University, Qingdao, P.R China; 3 The Key Laboratory of Hypertension, The Affiliated Hospital of Qingdao University, Qingdao, P.R China; 4 Graduate School of Medicine, Mie University, Mie, Japan; 5 TEDA International Cardiovascular Hospital, Tianjin & The Affiliated Hospital of Hangzhou Normal University, Hangzhou, P.R China; 6 Department of Surgery, Oregon Health and Science University, Portland, Oregon; 7 Medical College of Qingdao University, Qingdao, P.R China

## Abstract

The aim of our study was to evaluate the association between polymorphisms in the methylenetetrahydrofolate reductase (*MTHFR*) gene and the risk for congenital heart disease (CHD). Electronic literature databases were searched to identify eligible studies published before *Jun, 2014*. The association was assessed by the odds ratio (OR) with a 95% confidence interval (CI). The publication bias was explored using Begg's test. Sensitivity analysis was performed to evaluate the stability of the crude results. A total of 35 studies were included in this meta-analysis. For the *MTHFR* C677T polymorphism, we detected significant association in all genetic models for Asian children and the maternal population. Significant association was also detected in T vs. C for a Caucasian paediatric population (OR = 1.163, 95% CI: 1.008–1.342) and in both T vs. C (OR = 1.125, 95% CI: 1.043–1.214) and the dominant model (OR = 1.216, 95% CI:b1.096–1.348) for a Caucasian maternal population. For the *MTHFR* A1298C polymorphism, the association was detected in CC vs. AC for the Caucasian paediatric population (OR = 1.484, 95% CI: 1.035–2.128). Our results support the *MTHFR* -677T allele as a susceptibility factor for CHD in the Asian maternal population and the -1298C allele as a risk factor in the Caucasian paediatric population.

Congenital heart disease (CHD) is the most frequently occurring congenital disorder in newborns and is the most frequent cause of infant death from birth defects. The aetiology of CHD is largely unknown. Epidemiological studies reveal a significant environmental contribution to the pathogenesis of CHD^1^,^2^. Familial aggregation and twin studies indicate the presence of genetic factors for susceptibility to this condition^3^,^4^,^5^. Except for a few types of CHD induced by a single gene mutation, the majority of CHDs are polygenic diseases affected by both genetic and environmental factors.

The importance of genetic factors in the development of CHD is also supported by recent data from genome-wide association studies (GWASs). Data from these studies have confirmed that a region on chromosome 4p16 adjacent to the *MSX1* and *STX18* genes was associated with the risk of ostium secundum atrial septal defect (ASD)^6^, and rs2228638 in *NRP1* on 10p11 significantly increased the risk of Tetralogy of Fallot (TOF)^7^. In our studies, we identified *HOMEZ and PLAGL1* as pathogenic genes in Chinese patients with isolated ventricular septal defects (VSDs)^8^,^9^. In addition, our proteomic study revealed plasma protein changes in CHD patients^10^.

The 5,10-methylenetetrahydrofolate reductase (*MTHFR*) gene is located on chromosome 1 at 1p36.3. MTHFR is the key metabolic enzyme of homocysteine (Hcy). It catalyses 5,10-methylenetetrahydrofolate reduction to 5-methyltetrahydrofolate, which as a methyl donor induces Hcy remethylation to methionine^11^. A common C677T mutation (rs1801133) in the *MTHFR* gene has been described, which results in the conversion of the amino acid alanine to valine at position 226 in the protein. This mutation was associated with a 50% reduction of MTHFR enzyme activity, an increase in plasma Hcy concentration and a decrease in plasma folic acid concentration. Another polymorphism (A1298C, rs1801131) is located in exon 7, within the presumptive regulatory domain, and results in a glutamate-to-alanine change with decreased enzyme activity in vitro^12^. It has been reported that *MTHFR* polymorphisms play important roles in diseases. For example, neural tube defects and pregnancy complications appear to be linked to impaired MTHFR function^13^,^14^.

Since Wenstrom first noted an association between *MTHFR* gene polymorphism and susceptibility to CHD^15^, other studies have been undertaken to replicate this work. However, previous case-control reports have yielded inconsistent results. Wang and co-workers carried out a meta-analysis involving 2,554 CHD patients and 3,838 controls by searching the electronic literature for articles published before *July 22, 2012.* They suggested that the infant and maternal *MTHFR* C667T polymorphism may be associated with an increased occurrence of CHD^16^. By contrast, Mamasoula and co-workers indicated that the *MTHFR* C677T polymorphism, which directly influences plasma folate levels, is not associated with the risk of CHD^17^. Therefore, we performed an up-dated meta-analysis of all published studies (until *Jun, 2014*) to investigate the association between *MTHFR* polymorphisms (C677T and A1298C) and the risk of CHD.

## Methods

### Search strategy

We conducted a comprehensive search of Embase, Ovid, Web of Science, the Cochrane database, Medline (PubMed), the Chinese Biomedical Literature Database (CBM-disc, 1979–2014), the database of National Knowledge Infrastructure (CNKI, 1979–2014) and the full paper database of Chinese Science and Technology of Chongqing (VIP, 1989–2014) to identify suitable studies published before *Jun, 2014.* The following keywords were used for searching: (“congenital heart” OR “congenital cardiac” OR “heart defect*” OR “congenital car*”) AND (“polymorphism*” OR “variant*”) AND (“methylenetetrahydrofolate reductase” OR “MTHFR”). The most complete and recent results were used when there were multiple publications from the same study group. The references of reviews and retrieved articles were also searched simultaneously to find additional eligible studies.

### Inclusion criteria

Two investigators reviewed all identified studies independently to determine whether an individual study was eligible for inclusion. The selection criteria for studies to be considered for this meta-analysis were as follows: 1) *MTHFR* polymorphisms in CHD; 2) case-control or case-cohort study; 3) proper CHD diagnosis criteria; 4) original data; 5) human subjects, not animal studies. We expected the clinical assessment of the patients to include anthropometric measurement and physical examination for dysmorphism and malformation, and diagnostic studies to include chest X-ray examination, electrocardiogram, ultrasonic echocardiogram, etc. Studies would be excluded if the necessary information could not be obtained.

### Data extraction

Two investigators extracted the data independently, and a third investigator reviewed the result. The following information was extracted from each study: first author, year of publication, study population (country, ethnicity), the number of patients and controls in the study, genotype information, genotype methods, and main types of CHD. If any data essential to the analysis were not available from a study, best efforts were made to contact the authors to fill in the missing data.

### Statistical analysis

Allele frequencies for the *MTHFR* (C677T and A1298C) polymorphisms from each study were determined by the allele counting method^18^. The genotype distributions of controls were used to estimate the frequency of the putative risk allele (-677T and -1298C) using the inverse variance method^19^,^20^. The Hardy-Weinberg Equilibrium (*HWE*) is the most fundamental rule of population genetics. It prescribes the genotype frequencies at a locus in terms of its allele frequencies in a population. In the most general form, it states that selection, migration, and random genetic drift occur with random mating in a population in the absence of mutation^21^. The deviation from *HWE* for the distribution of the allele frequencies was analysed by Fisher's exact test in control groups. We examined the contrast of a vs. A, aa vs. AA, aa vs. Aa and also examined the recessive genetic model (aa vs. AA+Aa) and the dominant genetic model (Aa+aa vs. AA). The associations between *MTHFR* polymorphisms and CHD susceptibility were estimated by OR and its 95% CI. The significance of the pooled OR was determined by the Z-test; *P* < 0.05 was considered statistically significant. To evaluate the specific effects of ethnicity, stratified analyses were performed.

Heterogeneity across the eligible studies was tested using the Q-test, and the results were considered statistically significant when *P* < 0.1^22^,^23^. Heterogeneity was also quantified with the *I*^*2*^ metric (*I*^*2*^* = (Q - df)/Q × 100%*; *I*^*2*^ < 25%, no heterogeneity; *I*^*2*^ = 25–50%, moderate heterogeneity; *I*^*2*^ = 50–75%, large heterogeneity; *I*^*2*^ > 75%, extreme heterogeneity). When the effects were assumed to be homogenous (*P* > 0.1, *I*^*2*^ < 50%), the fixed-effects model was used; otherwise, the random-effects model was more appropriate^24^,^25^,^26^. Sensitivity analysis was performed to evaluate the stability of the results. If more than seven studies were included, Begg's test was used to measure publication bias, which was shown as a funnel plot^27^,^28^. *P* < 0.05 was considered representative of statistically significant publication bias. All analyses were performed using STATA software, version 10.0 (Stata Corporation, College Station, TX, USA), Review Manager (RevMan version 5.1.1, The Nordic Cochrane Centre: http://ims.cochrane.org/revman/download) and R statistical software (version 2.15.2, http://www.r-project.org).

## Results

### Studies included in the meta-analysis

**Figure 1:**
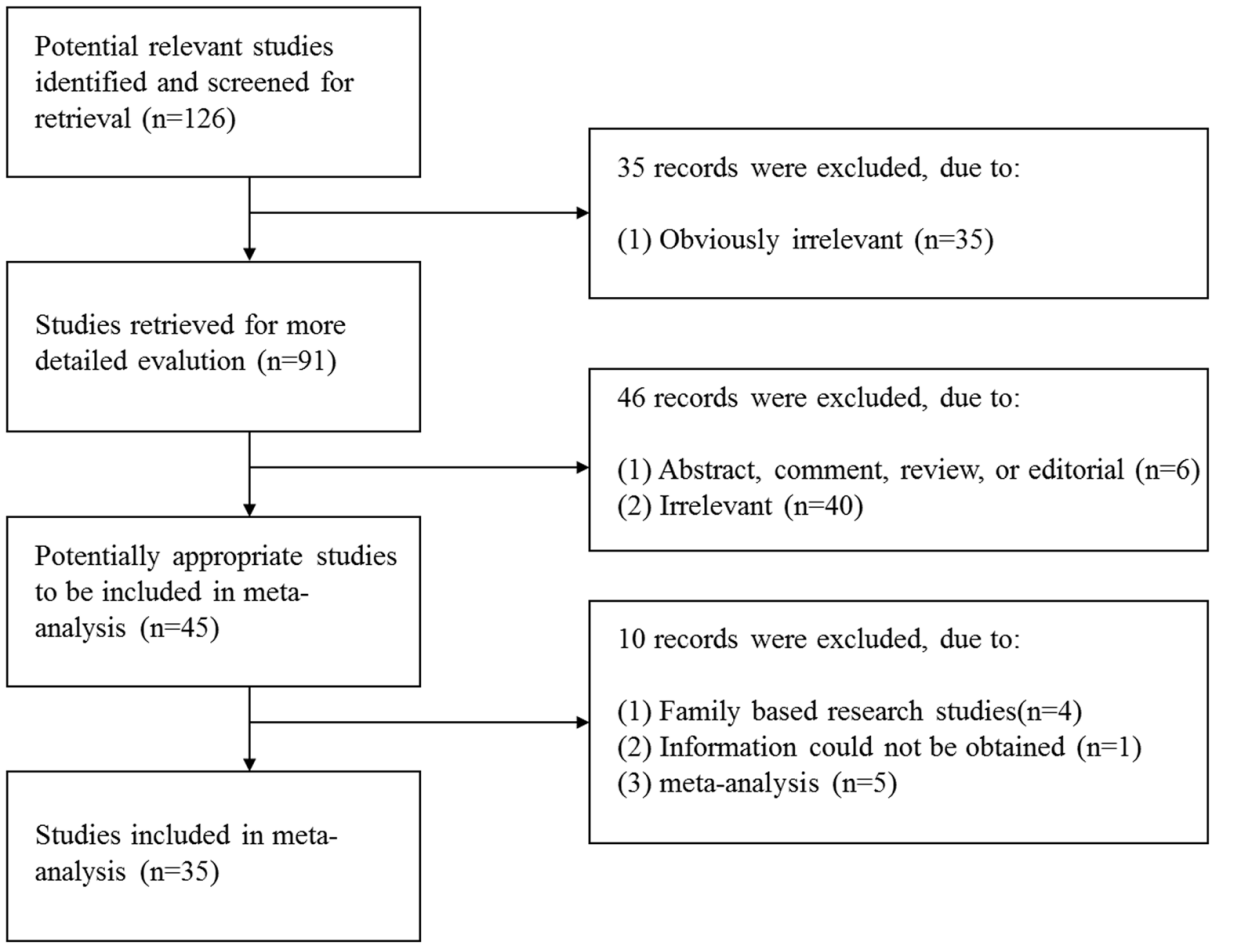
Flow chart of the study selection process and specific reasons for exclusion from the meta-analysis.

A total of 126 abstracts that met the inclusion criteria were retrieved through the databases. Two reviewers then selected the relevant studies independently. Forty-five relevant studies that described the association between the *MTHFR* polymorphism and CHD were identified. However, after reading the full articles and contacting the authors, we excluded five meta-analysis studies^29^,^30^,^31^,^32^,^33^, four family-based studies^34^,^35^,^36^,^37^, and one study in which information could not be obtained even after the authors were contacted^38^. Figure 1 shows the process of study selection and exclusion, with specification of reasons. Finally, 35 studies that met the inclusion criteria, corresponding to 9,329 CHD children and 15,076 normal controls, 3,232 mothers with CHD offspring and 27,174 normal controls for the C677T polymorphism and 1,761 CHD children and 1,868 normal controls/705 mothers with CHD offspring and 15,458 controls for the A1298C polymorphism, were considered in the meta-analysis^15^,^17^,^39^,^40^,^41^,^42^,^43^,^44^,^45^,^46^,^47^,^48^,^49^,^50^,^51^,^52^,^53^,^54^,^55^,^56^,^57^,^58^,^59^,^60^,^61^,^62^,^63^,^64^,^65^,^66^,^67^,^68^,^69^,^70^,^71^. The main characteristics of the included studies are listed in Table 1–2.

### Pooled Prevalence of *MTHFR* -677T and -1298C in the Controls

The pooled *MTHFR* –677T allele frequency determined using the random-effects model was 28.99% (95 CI: 26.14%–32.02%) in the Caucasian paediatric population and was 42.28% (95% CI: 34.17%–50.83%) in the Asian paediatric population. There was no heterogeneity among the Caucasian and Asian maternal population studies. The *MTHFR* –677T allele frequency was 31.76% (95 CI: 30.14%–33.43%) in the Caucasian maternal population and was 41.51% (95% CI: 37.50%–45.64%) in the Asian maternal population.

The pooled –1298C allele frequency in the fixed-effects model was 33.12% (95 CI: 29.80%–36.61%) in the Caucasian paediatric population and was 31.09% (95% CI: 25.34%–37.46%) in the Caucasian maternal population using the random-effects model.

### Association between *MTHFR* C677T polymorphism and risk of CHD

**Figure 2:**
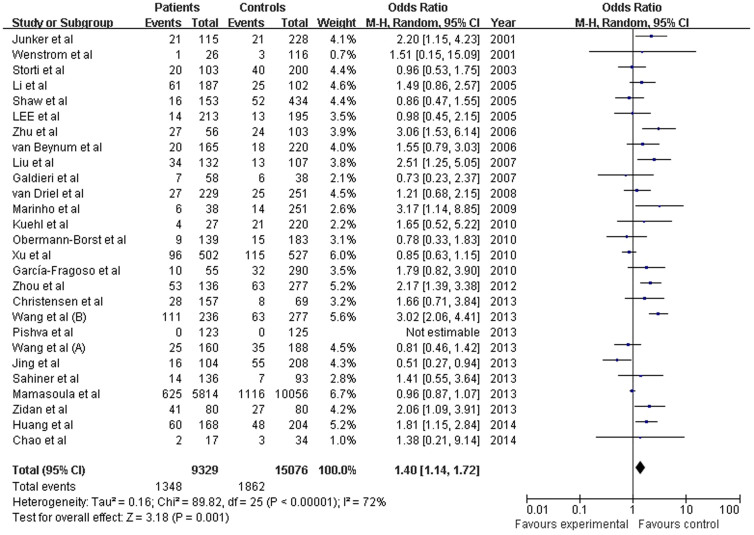
Pooled OR (recessive model) and 95% CI for individual studies and pooled data for the association between the polymorphism C677TT and congenital heart disease (CHD) in the overall paediatric population.

**Figure 3:**
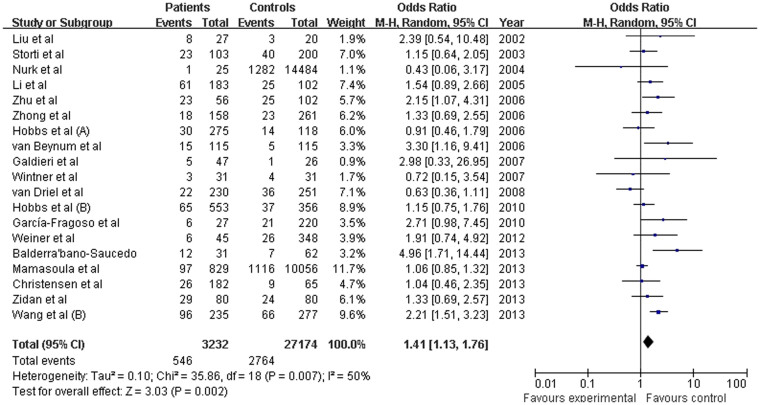
Pooled OR (recessive model) and 95% CI for individual studies and pooled data for the association between the polymorphism C677TT and congenital heart disease (CHD) in the overall maternal population.

We investigated the association between the *MTHFR* C677T polymorphism and the risk of CHD for each study. When all the eligible studies were pooled in the overall population of children with random-effects models, significant associations were observed in all genetic models: T versus C (OR = 1.248, 95% CI: 1.093–1.426; *P* = 0.001), TT versus CC (OR = 1.485, 95% CI: 1.140–1.935; *P* = 0.003), and TT versus CT (OR = 1.312, 95% CI: 1.100–1.565; *P* = 0.003), the dominant model (OR = 1.240, 95% CI: 1.053–1.461; *P* = 0.010), and the recessive model (OR = 1.410, 95% CI: 1.139–1.724; *P* = 0.001;(Figure 2). In addition, significant associations were observed in the overall maternal population in all genetic models for T versus C (OR = 1.215, 95% CI: 1.085–1.361; *P* = 0.001), TT versus CC (OR = 1.488, 95% CI: 1.169–1.859; *P* = 0.001), TT versus CT (OR = 1.315, 95% CI: 1.042–1.659; *P* = 0.021), the dominant model (OR = 1.258, 95% CI: 1.144–1.383; *P* = 2.14e-6), and the recessive model (OR = 1.408, 95% CI: 1.128–1.757; *P* = 0.002; (Figure 3). The Z-test indicated that the pooled ORs were statistically significant.

In the stratified analysis by ethnicity, significant associations were found when all studies were pooled with fixed or random-effects models for T versus C (OR = 1.163, 95% CI: 1.008–1.342; *P* = 0.039) in Caucasian children, and for T versus C (OR = 1.125, 95% CI: 1.043–1.214; *P* = 0.002), dominant model (OR = 1.216, 95% CI: 1.096–1.348; *P* = 2.24e-4) in the Caucasian maternal population. In addition, significant associations were found when all studies were pooled in fixed or random-effects models for all genetic models in Asian children and the maternal population. The main results of meta-analysis are shown in Table 3.

### Association between MTHFR A1298C polymorphism and risk of CHD

We investigated the association between the *MTHFR* A1298C polymorphism and the risk of CHD for each study. Overall, when all the eligible studies were pooled in the fixed-effects model, significant associations were observed for CC vs. AC (OR = 1.354, 95% CI: 1.022–1.793; *P* = 0.034), and for the recessive model (OR = 1.322, 95% CI: 1.015–1.732; *P* = 0.038) in the overall paediatric population. The main results of the meta-analysis are shown in Table 4.

**Figure 4:**
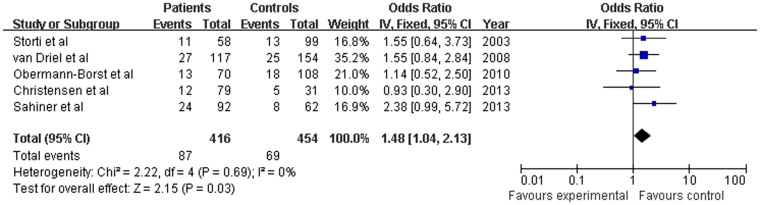
Pooled OR (CC vs. AC) and 95% CI of individual studies and pooled data for the association between the polymorphism A1298C and congenital heart disease (CHD) in the Caucasian paediatric population.

In the analysis stratified by ethnicity, significant associations were found in the Caucasian paediatric population when all studies were pooled in the fixed-effects model for CC versus AC (OR = 1.484, 95% CI: 1.035–2.128; *P* = 0.032; Figure 4). The main results of the meta-analysis are shown in Table 4.

### Sensitivity analyses

We removed the studies due to the genotype distribution in the control groups deviating from *HWE*. We found that the corresponding ORs for the C677T polymorphism for the TT vs. CT and recessive models in the overall paediatric population and for all genetic types in the overall maternal population and the Asian maternal population were not substantially altered (Table 5). This finding supports the reliability of the results.

### Publication bias

**Figure 5:**
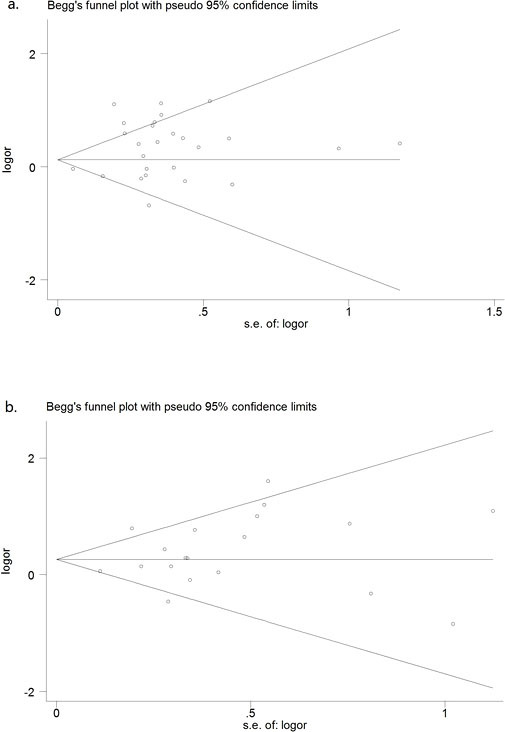
Funnel plot of the C1858T polymorphism and susceptibility to CHD (recessive model) in (a) the overall paediatric population (z = 0.18, *P* = 0.860) and (b) the overall maternal population (z = 0.91, *P* = 0.363).

Begg's test and a funnel plot were performed to assess the publication bias of the literature. We detected publication biases for the C677T polymorphism for the T vs. C and dominant models in the Caucasian paediatric population (Table 3). This might represent a limitation of our analysis because the studies with null findings, especially those with small sample size, were less likely to be published. By using the trim and fill method, we showed that, if the publication bias was the only source of the funnel plot asymmetry, they needed two and one more studies, respectively, to balance the funnel plot. The adjusted risk estimate was attenuated. The adjusted OR for T vs. C was 1.142 (95% CI: 0.729–1.786) and for the dominant model was 1.253 (95%CI: 0.738–2.133). The results suggest no evidence of publication biases in other genetic models and populations (Figure 5).

## Discussion

It is estimated that 7.9 million children are born with a serious birth defect of genetic or partially genetic origin each year in the world. CHDs are the most commonly occurring conditions. However, the aetiology of CHDs is largely unknown, and there are no established strategies for reducing their public health impact.

Many studies have demonstrated that genetic factors play important roles in the pathogenesis of CHD. In our previous studies, we have detected several novel variations of the *PLAGL1* and *HOMEZ* genes in Chinese patients with isolated VSD. We believe that these two genes are directly linked aetiologically with isolated VSD in the population^8^,^9^. In addition, the results of recent genome-wide association studies indicated that a region on chromosome 4p16 adjacent to the *MSX1* and *STX18* genes was associated (*P* = 9.5 × 10^−7^) with the risk of ostium secundum ASD^6^. These studies also showed that 1p12 (rs2474937 near *TBX15*; *P* = 8.44 × 10^−10^) and 4q31.1 (rs1531070 in *MAML3*; P = 4.99 × 10^−12^) were associated with congenital heart malformations in Han Chinese populations^72^.

In 1999, Kapusta and associates first reported that maternal hyperhomocysteinaemia is correlated with an increased risk of CHDs^73^. More recently, Hobbs and co-workers studied mothers whose pregnancies were affected by congenital heart defects (224 case subjects) or unaffected by any birth defect (90 control subjects) and identified Hcy, S-adenosylhomocysteine, and methionine as the most important biomarkers predictive of case or control status^36^. The MTHFR protein is a key enzyme in Hcy metabolism. The *MTHFR* gene is located on chromosome 1 at 1p36.3. The major product of the *MTHFR* gene is a catalytically active 77 kDa protein that catalyses the conversion of 5,10-methylenetetrahydrofolate into 5-methyltetrahydrofolate, the major circulating form of folate. Two common genetic polymorphisms associated with reduced MTHFR activity have been identified. The C677T polymorphism is located in exon 4 at the folate-binding site and results in an alanine-to-valine substitution. In healthy homozygous subjects, the 677TT genotype is associated with higher total Hcy and lower folate plasma level. The other polymorphism (A1298C) is in exon 7 within the presumptive regulatory domain and results in a glutamate-to-alanine change. Heterozygosity and homozygosity are associated neither with higher total Hcy nor lower folate plasma concentration. The *MTHFR* gene polymorphisms are directly linked with many diseases^20^,^74^. Our recent meta-analysis demonstrated that the *MTHFR* C677T polymorphism is associated with the risk of myocardial infarction in young/middle-aged Caucasians and is associated with susceptibility to preeclampsia^20^,^74^.

A number of studies have investigated the association between *MTHFR* genotype and the risk of CHD. In fact, in the last few years, several case–control studies were performed on this topic. However, the results are inconclusive. The two most recent meta-analyses for associations between polymorphism and CHD also led to conflicting conclusions. By reviewing all studies published before *April, 2011,* Yin and co-workers suggested that the foetal and paternal *MTHFR* C667T gene may be associated with an increased occurrence of CHD^32^. By contrast, after analysis of 7,698 cases and 13,159 controls by reviewing studies published before *2010*, Mamasoula and co-workers indicated that the same polymorphism, which directly influences plasma folate levels, is not associated with CHD risk^17^. Others also conducted meta-analysis to evaluate the association between MTHFR polymorphism and CHD^29^,^30^,^31^. It is possible that the relatively small sample size of these studies affected the accuracy of the results. Therefore, it is essential to re-perform a meta-analysis to evaluate the association. In our present study, we enlarged the sample size to 24,405 participants (9,329 CHD children and 15,076 normal controls), and performed sensitivity analysis to evaluate the stability of the results. In addition, we are the first to evaluate the association between the *MTHFR* A1298C polymorphism and CHD by meta-analysis. We are indebted to Dr. Christensen from McGill University for kindly allowing us access to his previously un-published data for this meta-analysis.

Our results indicate that the frequency of the putative risk allele -677T was 28.99% in Caucasian children and 31.76% in the Caucasian maternal population, whereas the frequency of -677T was 42.28% in Asian paediatric and 41.51% in the Asian maternal population. In addition, the pooled –1298C allele frequency was 33.12% in Caucasian children and 31.09% in the Caucasian maternal population. The meta-analysis results showed that associations exist between the *MTHFR* C677T polymorphism and susceptibility to CHD for all genetic models in all paediatric and maternal populations, especially in the Asian population. We also detected a significant association in the genetic model for T vs. C in the Caucasian paediatric population and in T vs. C and TT vs. CT for the Caucasian maternal population (Table 3). In our analysis of the A1298C polymorphism, we detected an association in the genetic model for TT vs. CT in the Caucasian paediatric population (Table 4). The results showing significant association for all genetic models in the overall maternal population and the Asian maternal population, and for the TT vs. CT and recessive models in the overall paediatric population were found to be stable and reliable by sensitivity analyses (Table 5).

Some limitations of this meta-analysis should be discussed. First, significant heterogeneity was observed in some genetic models when we pooled ORs. Under this condition, we used the random-effects model to pool the data. Sensitivity analysis was performed to evaluate the stability of the crude results. Second, publication biases appear to substantially contaminate the literature with regard to some genetic associations. The results of the trim and fill method demonstrated that the publication biases may affect the stability of positive results.

In conclusion, our results support the *MTHFR* –677T allele as a susceptibility factor for CHD in the Asian maternal population and the -1298C allele as a risk factor in the Caucasian paediatric population. Because of the heterogeneity and publication bias, we believe that other positive results may not be stable in our meta-analysis. A large number of homogeneous studies should be performed to evaluate these crude results in the future.

## Author Contributions

Conception and design of the study: C.X. and L.M.L. Acquisition of data: H.L., J.X.Z. and H.W.W. Analysis and interpretation of the data: C.X., H.L., J.X.Z., Y.W., C.P.N., Z.L. and B.B.Z. Writing and revision of the manuscript: C.X., L.M.L. G.W.H. All authors reviewed the manuscript.

## Figures and Tables

**Table 1:** The detailed characteristics of all eligible studies for *MTHFR* C677T polymorphism

			*MTHFR* C677T															
			Children							Mother								
			Cases			Controls				Cases			Controls					
Study	Year	Country (Ethnicity **)	CC	CT	TT	CC	CT	TT	*HWE*	CC	CT	TT	CC	CT	TT	*HWE*	Methods	Main types of CHD *</p>
Junker et al	2001	Germany (C)	52	42	21	129	78	21	0.087	—	—	—	—	—	—	—	PCR-RFLP	PS, HLHS, CoA, AVS, d-TGA, ASD, VSD, AVSD, TOF, PDA, DIV, PA, TA, Ebstein's Anomaly.
Wenstrom et al	2001	USA(90% C)	17	8	1	104	9	3	0.006	—	—	—	—	—	—	—	PCR-RFLP	HLV, HRV, CoA, PS, PA, TA, LVA, Atrioventricular Canal, Truncus Arteriosus, DORA, ASD, VSD.
Liu et al	2002	China (A)	—	—	—	—	—	—	—	5	14	8	2	15	3	0.068	PCR-RFLP	VSD ASD, TOF, PDA, Single Atrium/Ventricle
Storti et al	2003	Italy (C)	28	55	20	52	108	40	0.259	27	53	23	52	108	40	0.259	PCR-RFLP	VSD,TOF, DORV, PA, d-TGA, AC
Nurk et al	2004	Norway (C)	—	—	—	—	—	—	—	12	12	1	7165	6037	1282	0.842	RT-PCR	Congenital Anomalies Heart
Li et al	2005	China (A)	32	94	61	20	57	25	0.320	32	90	61	20	57	25	0.320	PCR-RFLP	VSD, ASD, PDA, TOF,
LEE et al	2005	China (A)	110	89	14	114	68	13	0.556	—	—	—	—	—	—	—	PCR-DHPLC	AP Window, ASD, CoA, PS, DILV, DORV, ECD, IAA, LAI, PA, PDA, RAI, TGA, TOF, VSD.
Shaw et al	2005	USA (C)	69	68	16	180	202	52	0.753	—	—	—	—	—	—	—	ARRAY	TOF, d-TGA, Truncus Arteriosus, DORV, PA, VSD, AP-Window
Hobbs et al	2006	USA (C)	—	—	—	—	—	—	—	127	118	30	48	56	14	0.841	SEQUENCE	Nonsyndromic Septal, Conotruncal, or right- or left-sided ObstructiveHeart Defect
Zhu et al	2006	China (A)	7	22	27	22	57	24	0.328	6	27	23	20	57	25	0.320	PCR-RFLP	ASD, PDA
Zhong et al	2006	China (A)	—	—	—	—	—	—	—	67	33	15	76	34	5	0.558	PCR-RFLP	Congenital Heart Disease
van Beynum et al	2006	Netherlands (C)	79	66	20	98	104	18	0.216	72	68	18	131	107	23	0.881	PCR-RFLP	TOF, VSD, Truncus Arteriosus, TGA, AP-Window, TVA, AVSD, PS, AS, HLHS, CoA, PDA,
Galdieri et al	2007	Brazil (M)	30	21	7	18	14	6	0.286	27	15	5	10	15	1	0196	PCR-RFLP	Congenital Heart Defects
Wintner et al	2007	Austria (C)	—	—	—	—	—	—	—	16	12	3	10	17	4	0.708	ARRAY	TOF,HLHS, TGA, DORV, VSD, AS, CoA, PS, Anomalies of the Aortic Arch
Liu et al	2007	China (A)	30	68	34	46	48	13	0.829	—	—	—	—	—	—	—	PCR-RFLP	Congenital Heart Disease
van Driel et al	2008	Netherlands(C)	99	103	27	119	107	25	0.884	91	117	22	111	104	36	0.166	PCR-RFLP	TOF, TGA, ASD, VSD, CoA, AS, PS, HLHS,
Marinho et al	2009	Portugal (M)	12	20	6	113	124	14	0.073	—	—	—	—	—	—	—	PCR-RFLP	TOF
Obermann-Borst et al	2010	Netherlands (C)	64	66	9	92	76	15	1.000	—	—	—	—	—	—	—	PCR-RFLP	TOF, TGA, ASD, VSD, CoA, AS, PS, HLHS
Hobbs et al	2010	USA (C)	—	—	—	—	—	—	—	285	203	65	191	128	37	0.036	SEQUENCE	Nonsyndromic Septal, Conotruncal, or Right- or Left-sided ObstructiveHeart Defect
Xu et al	2010	China (A)	162	244	96	151	261	115	0.930	—	—	—	—	—	—	—	PCR-RFLP	Cyanotic Cardiac Disease, ASD, VSD, PDA, Left-sided Obstruction Defects
García-Fragoso et al	2010	Puerto Rico (M)	9	14	4	84	115	21	0.056	10	11	6	84	115	21	0.056	PCR-RFLP	HLHS, TOF, DORV, TGA, VSD, PS, AS, CoA, ASD, Ebstein's Anomaly.
Kuehl et al	2010	USA (C)	12	33	10	134	124	32	0.688	—	—	—	—	—	—	—	ARRAY	CoA
Weiner et al	2012	Russia (C)	—	—	—	—	—	—	—	18	21	6	173	149	26	0.514	RT-PCR	Congenital Anomalies-cardiovascular System
Zhou et al	2012	China (A)	23	60	53	88	126	63	0.183	—	—	—	—	—	—	—	PCR-RFLP	TOF
Pishva et al	2013	Malaysia (SA)	63	60	0	71	54	0	0.001	—	—	—	—	—	—	—	PCR-RFLP	VSD
Mamasoula et al	2013	UK(M)	2759	2430	625	4826	4114	1116	0.000	336	396	97	4826	4114	1116	0.000	SEQUENCE	Congenital Heart Disease
Wang et al	2013	China(A)	59	76	25	53	100	35	0.377	—	—	—	—	—	—	—	SEQUENCE	Congenital Heart Disease
Jing et al	2013	China (A)	46	42	16	39	114	55	0.164	—	—	—	—	—	—	—	PCR-RFLP	Congenital Heart Disease
Sahiner et al	2013	Turkey(C)	69	53	14	47	39	7	1.000	—	—	—	—	—	—	—	PCR-RFLP	Obstruction in LV Output, Left-to-right Shunt, Conotruncal Anomalies, Complex Anomalies
Zidan et al	2013	Egypt (AR)	18	21	41	32	21	27	0.000	21	30	29	31	25	24	0.001	PCR-RFLP	ASD, VSD, PDA, PS, TOF, HLHS, Combined Lesion
Balderra' bano-Saucedo et al	2013	Mexico (M)	—	—	—	—	—	—	—	7	12	12	24	31	7	0.595	PCR-RFLP	Complex Congenital Heart Disease
Christensen et al ***	2013	USA (C)	68	61	28	35	26	8	0.395	67	89	26	27	29	9	0.791	PCR-RFLP	VSD, TOF, AS, TGA, AVSD, DORV, PS, CoA, Truncus Arteriosus
Wang et al	2013	China (A)	33	92	111	88	126	63	0.183	39	100	96	82	129	66	0.279	PCR-RFLP	VSD, ASD, PDA, TOF, DORV
Huang et al	2014	China (A)	63	45	60	84	72	48	0.000	—	—	—	—	—	—	—	MASS SPECTRUM	TOF
Chao et al	2014	China (A)	10	5	2	19	12	3	0.660	—	—	—	—	—	—	—	PCR-RFLP	PDA

*: PS: Pulmonary Stenosis; **HLHS**: Hypoplastic Left Heart Syndrome; **CoA**: Coarctation of the Aorta; **AVS**: Aortic Valve Stenosis; **TGA**: Transposition of Great Arteries; **ASD**: Atrial Septal Defect; **VSD**: Ventricular Septal Defect; **AVSD**: Atrioventricular Septal Defect; **TOF**: Tetralogy of Fallot; **PDA**: Patent Ductus Arteriosus; **DIV**: Double Inlet Ventricle; **PA**: Pulmonary Atresia; **TA**: Tricuspid Atresia; **HLV**: Hypoplastic Left Ventricle; **HRV**: Hypoplastic Right Ventricle; **LVA**: Left Ventricular Aneurysm; **DORV**: Double-outlet Right Ventricle; **AC**: Aortic Coarctation; **AP window**: Atriopulmonary window; **ECD**: Endocardial Cushion Defect; **IAA**: Interrupted Aortic Arch; **LAI**: Left Atrial Isomerism; **RAI**: Right Atrial Isomerism; **TVA**: Tricuspid Valve Atresia; **AS:** Aortic Stenosis.

**: C: Caucasians; A: South Asians; M: Mixed; AR: Arabian.

***: The data was respectively provided by author of Dr. **Karen E. Christensen** (see Acknowledgements).

**Table 2:** The detailed characteristics of all eligible studies for *MTHFR* A1298C polymorphism

			*MTHFR* A1298C															
			Children							Mother								
			Cases			Controls				Cases			Controls					
Study	Year	Country (Ethnicity **)	AA	AC	CC	AA	AC	CC	*HWE*	AA	AC	CC	AA	AC	CC	*HWE*	Methods	Main types of CHD *
Storti et al	2003	Italy (C)	45	47	11	101	86	13	0.387	49	46	8	101	86	13	0.387	PCR-RFLP	VSD,TOF, DORV, PA, d-TGA, AC
Nurk et al	2004	Norway (C)	—	—	—	—	—	—	—	9	13	3	6607	6342	1525	0.955	RT-PCR	Congenital Anomalies Heart
Galdieri et al	2007	Brazil (M)	35	21	1	19	16	3	1.000	26	17	4	15	10	1	1.000	PCR-RFLP	Congenital Heart Defects
van Driel et al	2008	Netherlands (C)	112	90	27	97	129	25	0.073	104	102	24	116	104	31	0.319	PCR-RFLP	TOF, TGA, ASD, VSD, CoA, AS, PS, HLHS,
Obermann-Borst et al	2010	Netherlands (C)	69	57	13	75	90	18	0.256	—	—	—	—	—	—	—	PCR-RFLP	TOF, TGA, ASD, VSD, CoA, AS, PS, HLHS
Xu et al	2010	China (A)	316	168	18	326	185	16	0.110	—	—	—	—	—	—	—	PCR-RFLP	Cyanotic Cardiac Disease, ASD, VSD, PDA, Left-sided Obstruction Defects.
Weiner et al	2012	Russia (C)	—	—	—	—	—	—	—	33	13	2	168	152	42	0.403	RT-PCR	Congenital Anomalies-cardiovascular System
Wang et al	2013	China (A)	115	45	10	133	47	8	0.186	—	—	—	—	—	—	—	SEQUENCE	Congenital Heart Disease
Sahiner et al	2013	Turkey (C)	45	68	24	31	54	8	0.029	—	—	—	—	—	—	—	PCR-RFLP	Obstruction in LV Output, Left-to-right Shunt, Conotruncal Anomalies, Complex Anomalies
Zidan et al	2013	Egypt (AR)	16	27	37	26	24	27	0.001	13	32	25	33	25	22	0.001	PCR-RFLP	ASD, VSD, PDA, PS, TOF, HLHS, Combined lesion
Christensen et al ***	2013	USA (C)	78	67	12	38	26	5	0.764	98	71	13	36	22	7	0.220	PCR-RFLP	VSD, TOF, AS, TGA, AVSD, DORV, PS, CoA, Truncus Arteriosus
Huang et al	2014	China (A)	111	56	3	146	56	6	0.800	—	—	—	—	—	—	—	MS	TOF

*: PS: Pulmonary Stenosis; **HLHS**: Hypoplastic Left Heart Syndrome; **CoA**: Coarctation of the Aorta; **TGA**: Transposition of Great Arteries; **ASD**: Atrial Septal Defect; **VSD**: Ventricular Septal Defect; **AVSD**: Atrioventricular Septal Defect; **TOF**: Tetralogy of Fallot; **PDA**: Patent Ductus Arteriosus; **PA**: Pulmonary Atresia; **DORV**: Double-outlet Right Ventricle; **AC**: Aortic Coarctation; **AS:** Aortic Stenosis.

**: C: Caucasians; A: South Asians; M: Mixed; AR: Arabian.

***: The data was respectively provided by author of Dr. **Karen E. Christensen** (see Acknowledgements).

**Table 3:** Main results of association between *MTHFR* C677T polymorphism and CHD

		Sample size		Test of heterogeneity			Test of association				Test of publication bias	
Subgroup	Genetic model	Patients	Controls	Q	*P*	I^2^ (%)	OR	95% CI	Z	*P*	z	*P*
**Children Overall**	T vs. C	9,329	15,076	146.67	0.000	82.3	**1.248**	**1.093–1.426**	**3.27**	**0.001**	1.13	0.260
	TT vs. CC			118.35	0.000	78.9	**1.485**	**1.140–1.935**	**2.93**	**0.003**	0.48	0.628
	TT vs. CT			53.62	0.001	53.4	**1.312**	**1.100–1.565**	**3.02**	**0.003**	0.66	0.508
	Dominant model			102.79	0.000	74.4	**1.240**	**1.053–1.461**	**2.58**	**0.010**	1.54	0.123
	Recessive model			89.82	0.000	72.2	**1.401**	**1.139–1.724**	**3.19**	**0.001**	0.18	0.860
**Maternal Overall**	T vs. C	3,232	2,7174	34.32	0.011	47.6	**1.215**	**1.085–1.361**	**3.38**	**0.001**	0.35	0.726
	TT vs. CC			32.94	0.017	45.4	**1.488**	**1.169–1.895**	**3.23**	**0.001**	1.33	0.174
	TT vs. CT			35.13	0.009	48.8	**1.315**	**1.042–1.659**	**2.31**	**0.021**	0.98	0.327
	Dominant model			25.69	0.107	29.9	**1.258**	**1.144–1.383**	**4.74**	**2.14e-6**	0.70	0.484
	Recessive model			35.86	0.007	49.8	**1.408**	**1.128–1.757**	**3.03**	**0.002**	0.91	0.363
**Caucasian Children**	T vs. C	7,092	12,150	26.94	0.003	62.9	**1.163**	**1.008–1.342**	**2.06**	**0.039**	2.18	**0.029**
	TT vs. CC			18.09	0.073	44.7	1.273	0.978–1.658	1.79	0.073	0.93	0.350
	TT vs. CT			9.29	0.505	0.0	0.986	0.892–1.090	0.28	0.781	0.62	0.533
	Dominant model			24.13	0.007	58.6	1.182	0.982–1.422	1.77	0.077	2.34	**0.020**
	Recessive model			13.12	0.217	23.8	1.012	0.921–1.113	0.26	0.798	0.47	0.640
**Caucasian Maternal**	T vs. C	2,431	26,170	9.22	0.417	2.4	**1.125**	**1.043–1.214**	**3.04**	**0.002**	0.89	0.371
	TT vs. CC			7.25	0.611	0.0	1.157	0.977–1.370	1.69	0.690	0.72	0.474
	TT vs. CT			6.95	0.643	0.0	0.945	0.800–1.116	0.67	0.504	0.00	1.00
	Dominant model			11.03	0.274	18.4	**1.216**	**1.096–1.348**	**3.69**	**2.24e-4**	0.54	0.592
	Recessive model			6.58	0.681	0.0	1.074	0.894–1.227	0.57	0.566	0.54	0.592
**Asian Children**	T vs. C	1,911	2,222	74.39	0.000	86.6	**1.449**	**1.117–1.880**	**2.79**	**0.005**	0.16	0.876
	TT vs. CC			62.4	0.000	83.9	**1.960**	**1.203–3.192**	**2.70**	**0.007**	0.16	0.876
	TT vs. CT			31.57	0.000	68.3	**1.649**	**1.209–2.248**	**3.16**	**0.002**	0.47	0.640
	Dominant model			43.94	0.000	77.2	**1.441**	**1.049–1.978**	**2.26**	**0.024**	0.16	0.876
	Recessive model			49.87	0.000	79.9	**1.761**	**1.227–2.526**	**3.07**	**0.002**	0.62	0.533
**Asian Maternal**	T vs. C	467	616	3.96	0.412	0.0	**1.595**	**1.348–1.886**	**5.45**	**5.04e-8**	–	–
	TT vs. CC			3.49	0.479	0.0	**2.548**	**1.788–3.631**	**5.18**	**2.22e-7**	–	–
	TT vs. CT			1.51	0.825	0.0	**1.884**	**1.415–2.509**	**4.34**	**1.42e-5**	–	–
	Dominant model			4.93	0.295	18.9	**1.605**	**1.215–2.121**	**3.33**	**0.001**	–	–
	Recessive model			2.05	0.727	0.0	**2.073**	**1.583–2.716**	**5.29**	**1.22e-7**	–	–

**Table 4:** Main results of association between *MTHFR* A1298C polymorphism and CHD

		Sample size		Test of heterogeneity			Test of association				Test of publication bias	
Subgroup	Genetic model	Patients	Controls	Q	*P*	I^2^ (%)	OR	95% CI	Z	*P*	z	*P*
**Children Overall**	C VS. A	1,834	1,744	14.21	0.077	43.7	1.044	0.890–1.225	0.53	0.595	0.31	0.754
	CC vs. AA			9.05	0.338	11.6	1.260	0.950–1.671	1.60	0.109	0.10	0.917
	CC vs. AC			4.56	0.804	0.00	**1.354**	**1.022–1.793**	**2.11**	**0.034**	1.56	0.118
	Dominant model			14.34	0.073	44.2	0.978	0.792–1.206	0.21	0.832	0.36	0.175
	Recessive model			5.83	0.666	0.0	**1.322**	**1.015–1.732**	**2.07**	**0.038**	0.52	0.602
**Maternal Overall**	C VS. A	705	15,458	16.60	0.011	63.9	1.041	0.781–1.386	0.27	0.785	0.60	0.548
	CC vs. AA			11.15	0.084	46.2	1.085	0.631–1.864	0.29	0.769	0.00	1.000
	CC vs.AC			2.07	0.913	0.0	0.841	0.587–1.205	0.94	0.346	0.60	0.548
	Dominant model			17.61	0.007	65.9	1.107	0.748–1.639	0.51	0.612	0.60	0.548
	Recessive model			5.39	0.495	0.00	0.966	0.690–1.352	0.20	0.839	0.30	0.764
**Caucasian Children**	C VS. A	765	796	6.76	0.149	40.8	0.989	0.848–1.154	0.14	0.891	–	–
	CC vs. AA			4.15	0.386	3.60	1.177	0.819–1.691	0.88	0.378	–	–
	CC vs. AC			2.22	0.695	0.00	**1.484**	**1.035–2.128**	**2.15**	**0.032**	–	–
	Dominant model			7.83	0.098	48.9	0.916	0.681–1.231	0.58	0.559	–	–
	Recessive model			2.92	0.571	0.00	1.332	0.944–1.878	1.63	0.103	–	–
**Caucasian Maternal**	C VS. A	588	15,352	9.17	0.057	56.4	0.920	0.693–1.223	0.57	0.567	–	–
	CC vs. AA			4.43	0.364	7.4	0.850	0.565–1.278	0.78	0.434	–	–
	CC vs. AC			1.25	0.870	0.00	0.802	0.531–1.212	1.05	0.295	–	–
	Dominant model			9.22	0.056	56.6	0.943	0.652–1.363	0.31	0.753	–	–
	Recessive model			2.77	0.597	0.00	0.824	0.557–1.217	0.97	0.330	–	–

**Table 5:** Sensitivity analysis of association between *MTHFR* C677T polymorphism and CHD

		Test of heterogeneity			Test of association			
Subgroup	Genetic model	Q	*P*	I^2^ (%)	OR	95% CI	Z	*P*
**Children Overall**	TT vs. CT	32.42	0.020	44.5	**1.303**	**1.064–1.596**	**2.56**	**0.010**
	Recessive model	61.61	0.000	70.8	**1.335**	**1.028–1.735**	**2.16**	**0.030**
**Maternal Overall**	T vs. C	32.48	0.006	53.8	**1.215**	**1.042–1.425**	**2.48**	**0.013**
	TT vs. CC	29.99	0.012	50.0	**1.570**	**1.125–2.192**	**2.65**	**0.008**
	TT vs. CT	26.09	0.037	42.5	**1.462**	**1.104–1.937**	**2.65**	**0.008**
	Dominant model	22.10	0.105	32.1	**1.198**	**1.035–1.386**	**2.43**	**0.015**
	Recessive model	29.49	0.014	49.1	**1.527**	**1.149–2.030**	**2.92**	**0.004**
**Asian Maternal**	T vs. C	3.96	0.412	0.0	**1.595**	**1.348–1.886**	**5.45**	**5.04e-8**
	TT vs. CC	3.49	0.479	0.0	**2.548**	**1.788–3.631**	**5.18**	**2.22e-7**
	TT vs. CT	1.51	0.825	0.0	**1.884**	**1.415–2.509**	**4.34**	**1.42e-5**
	Dominant model	4.93	0.295	18.9	**1.605**	**1.215–2.121**	**3.33**	**0.001**
	Recessive model	2.05	0.727	0.0	**2.073**	**1.583–2.716**	**5.29**	**1.22e-7**
